# Changes in P2Y_6_ receptor‐mediated vasoreactivity following focal and global ischemia

**DOI:** 10.14814/phy2.15283

**Published:** 2022-04-24

**Authors:** André Erdling, Sara Ellinor Johansson, Aneta Radziwon‐Balicka, Saema Ansar, Lars Edvinsson

**Affiliations:** ^1^ 70590 Department of Clinical Sciences Division of Experimental Vascular Research Lund University Lund Sweden; ^2^ Department of Cardiothoracic Surgery, Anesthesiology and Intensive Care Skane University Hospital Lund Sweden; ^3^ 70590 Applied Neurovascular Research Department of Clinical Sciences Lund University Lund Sweden; ^4^ Department of Clinical Experimental Research Glostrup Research Institute Rigshospitalet‐Glostrup Glostrup Denmark

**Keywords:** MCAO, P2Y6, purinergic, rat, SAH, stroke

## Abstract

Ischemia, both in the form of focal thromboembolic stroke and following subarachnoid hemorrhage (SAH), causes upregulation of vasoconstrictive receptor systems within the cerebral vasculature. Descriptions regarding changes in purinergic signaling following ischemia are lacking, especially when the importance of purinergic signaling in regulating vascular tone is taken into consideration. This prompted us to evaluate changes in P2Y_6_‐mediated vasomotor reactivity in two different stroke models in rat. We used wire myography to measure changes in cerebral vasoreactivity to the P2Y_6_ agonist UDP‐β‐S following either experimental SAH or transient middle cerebral artery occlusion. Changes in receptor localization or receptor expression were evaluated using immunohistochemistry and quantitative flow cytometry. Transient middle cerebral artery occlusion caused an increase in Emax when compared to sham (233.6 [206.1–258.5]% vs. 161.1 [147.1–242.6]%, *p* = 0.0365). No such change was seen following SAH. Both stroke models were associated with increased levels of P2Y_6_ receptor expression in the vascular smooth muscle cells (90.94 [86.99–99.15]% and 93.79 [89.96–96.39]% vs. 80.31 [70.80–80.86]%, *p* = 0.021) and *p* = 0.039 respectively. There was no change in receptor localization in either of the stroke models. Based on these findings, we conclude that focal ischemic stroke increases vascular sensitivity to UDP‐β‐S by upregulating P2Y_6_ receptors on vascular smooth muscle cells while experimental SAH did not induce changes in vasoreactivity in spite of increased P2Y_6_ receptor expression.

## Graphical abstract

We demonstrate the increased expression of vasoconstrictive P2Y6 receptors on the smooth muscle cells of rat middle cerebral arteries after transient middle cerebral artery occlusion (MCAO) and experimental subarachnoid hemorrhage (SAH). This is associated with an enhanced response to the P2Y6 receptor agonist UDP‐beta‐S following transient MCAO but not after SAH.

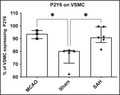

## 1 INTRODUCTION

There are several types of stroke in man, of which thromboembolic focal ischemic stroke is the most prevalent (about 80%) while the rupture of a brain artery resulting in subarachnoid hemorrhage (SAH) constitutes about 5%. Both forms of stroke cause a substantial amount of mortality and morbidity among the adult population in the developed world. Despite advances in diagnosis and treatment of SAH, effective pharmacological therapeutic interventions are still limited (Ciurea et al., 2013; Fujii et al., 2013). Survival and recovery after focal ischemic stroke have been greatly improved by therapeutic options such as thrombolysis and thrombectomy, but are still associated with increased mortality and disability (Brønnum‐Hansen et al., 2001). We have previously demonstrated early activation of the Ras‐Raf‐MEK‐ERK1/2 pathway in the cerebrovascular smooth muscle cells in both types of stroke and have demonstrated it to be an important pathway involved in cerebral vasculopathy after experimental SAH (Edvinsson & Povlsen, 2011) and transient middle cerebral artery occlusion (tMCAO; Henriksson et al., 2007). Cerebral vasculopathy involves multiple factors including vasospasm, vascular inflammation, and upregulation of vasoconstrictor receptors in the vessel wall that may worsen the outcome and cause further disability (Beg et al., 2006; Edvinsson & Povlsen, 2011; Maddahi et al., 2012; Vergouwen et al., 2011).

Changes in receptor systems favoring cerebral vasoconstriction, such as increased expression of the endothelin A (ET_A_), endothelin B (ET_B_), angiotensin 1 (AT_1_), and serotonin 1B (5‐HT_1B_) receptors following either SAH or thromboembolic stroke have been described (Edvinsson & Povlsen, 2011; Johansson et al., 2012; Müller et al., 2017) and can contribute to cerebral hypoperfusion and poor outcome. Inhibition of the MEK‐ERK1/2 pathway prevented the upregulation of these contractile receptors and improved functional outcomes and restored perfusion (Henriksson et al., 2007; Johansson et al., 2014; Mostajeran et al., 2018; Povlsen & Edvinsson, 2015).

Extracellular nucleotides are released in the extracellular space in response to stimuli such as shear stress, hypoxia or cell lysis, and act in an auto‐ or paracrine manner. Nucleotides may also be released as co‐transmitters from sympathetic and parasympathetic nerves or from circulating platelets or red blood cells (Ralevic & Dunn, 2015). Nucleotide effects are exerted through either ionotropic P2X‐receptors or through G‐protein coupled P2Y‐receptors. Purinergic signaling is important in the maintenance of vascular tone where purinoreceptors on the vascular endothelium induce vasodilation (Ralevic & Dunn, 2015; Wihlborg et al., 2003) while receptors located on smooth muscle cells favor vasoconstriction (Burnstock, 2017; Ralevic & Dunn, 2015). This leads to a dual purinergic control of vascular tone via perivascular nerves and endothelium.

The expression of nucleotide receptors varies between vascular beds and species. P2Y_6_ receptors are, for instance, expressed on both endothelial cells and smooth muscle cells of human arteries while rat mesenteric and cerebral arteries lack endothelial P2Y_6_ receptors (Burnstock, 2017; MaassenVanDenBrink et al., 2016; Wang et al., 2002).

The primary ligand for the P2Y_6_ receptor is UDP and to a much lesser extent UTP. Adenine nucleotides are more or less inactive. The receptor is coupled to G_q_ and activation induces signaling via the inositol triphosphate pathway through phospholipase C, resulting in the release of intracellular storages of Ca^2+^ (Kügelgen, 2019; Kügelgen & Hoffmann, 2016). The scarcity of data describing the potential changes in purinergic receptor systems following cerebrovascular insults prompted us to investigate the response to UDP‐β‐S, a potent P2Y_6_ receptor agonist, in the rat middle cerebral artery following either experimental transient middle cerebral artery occlusion (tMCAO) or experimental SAH. We hypothesize that cerebral ischemia induces increased expression of P2Y_6_ receptors on the vascular smooth muscle cells (VSMCs) as a response to decreased luminal pressure caused by either clot formation or global hypoperfusion.

## 2 MATERIAL AND METHODS

### 2.1 Experimental transient MCA occlusion

Male Wistar rats (340 [314–359] g) were anesthetized with isoflurane (2.5%–3% during induction, 2% during the procedure) in a 70:30 mixture of nitrous oxide and oxygen while maintaining spontaneous breathing. Arterial blood pressure and blood gases were measured through a tail artery catheter. Rectal temperature was monitored, and a thermic blanket aided in keeping body temperature at 37 °C during the procedure. A small hole was drilled in the cranium (1 mm posterior to the bregma and 6 mm to the right of the midline) and a laser‐doppler probe (Oxford Optronics) was fixed to the dura mater in order to monitor cerebral blood flow (CBF).

An incision in the midline of the neck was made, exposing the right internal, external, and common carotid arteries as described in detail previously (Prunell et al., 2002). The external and common carotid arteries were ligated before a coated monofilament (Doccol Corporation) was inserted in and advanced along the common carotid artery until the tip reached and occluded the proximal part of the right MCA. Adequate occlusion was confirmed by an abrupt drop in the laser‐doppler signal. Failure to position the filament or a reduction in CBF of less than 60% excluded the animal from further participation. Two animals with marginal drop were included after TTC (2,3,5‐triphenyltetrazolium chloride) staining and clinical data suggested large ischemic areas in brain areas supported by the MCA. Anesthesia was discontinued after the removal of the tail artery catheter and after subcutaneous administration of bupivacaine (1.25 mg/kg) in all open wounds. All rats were re‐anesthetized 2 hours after successful MCA occlusion and the occluding filament was removed allowing reperfusion which was confirmed by an immediate increase of CBF and laser‐doppler signal.

### 2.2 SAH and sham

SAH was induced as described in a previous paper (Povlsen & Edvinsson, 2015). In short, male Sprague‐Dawley rats (334 [310–376] g) were anesthetized with either a 2.5 mL·kg^−1^ mixture of fentanyl citrate (0.315 mg/ml), fluanisone (10 mg/ml) and midazolam (5 mg/ml) or either isoflurane or halothane in a 70:30 mixture of nitrous oxide and oxygen. The rats were mechanically ventilated and arterial blood pressure and arterial blood gases were monitored through a tail artery catheter. Body temperature was measured rectally and maintained at +37°C throughout the procedure by using a thermal blanket. A laser‐doppler probe (Oxford Optronics) was attached to the dura through a drilled hole in the cranium (4 mm anterior to the bregma and 3 mm to the right of the midline) in order to monitor CBF. Intracranial pressure (ICP) was monitored continuously through a catheter placed in the cisterna magna. A 27G blunt cannula was advanced stereotactically to a position immediate anterior of the chiasma opticum through a second hole. After 30 min of equilibration, 250–300 μl of blood was withdrawn from the tail catheter and injected manually through the cannula, aiming to increase ICP to the mean arterial blood pressure level. At the end of surgery and 24 h thereafter, rats received subcutaneous injections of Carprofen (4 mg/kg, a dose which has been shown not to abolish vascular inflammation after SAH (Maddahi et al., 2012; Povlsen et al., 2013)). A fluid bolus of normal saline was administered upon completion of surgery. Sham‐operated rats (335 [324–376] g) went through the same procedure with the exception that no blood was injected intracisternally.

### 2.3 Drugs

Uridine 5′‐O‐thiodiphosphate (UDP‐β‐S), a potent and selective P2Y_6_ receptor agonist (Hou et al., 2002; Malmsjö et al., 2003; Jena Bioscience) was dissolved in 0.9% saline to form a stock solution of 10^−2^ M. The stock solution was stored at −20°C until utilized. All other reagents were obtained from Sigma‐Aldrich unless otherwise stated in the text. All drug concentrations expressed indicate the final molar concentration in the myograph bath.

### 2.4 In vitro pharmacology

All rats were anesthetized with CO_2_ and euthanized 48 h after surgery. The brains were immediately removed and placed in a cold buffer where the MCAs were carefully isolated from the brain. The arteries were cut into 1–2 mm long segments and mounted in a wire myograph system (Danish Myograph Technology A/S) which was used to measure and record isometric tension. The vessel segments were submerged in a phosphate buffer solution (119 mM NaCl, 15 mM NaHCO_3_, 4.6 mM KCl, 1.2 mM MgCl_2_, 1.2 mM NaH_2_PO_4_, 1.5 mM CaCl_2_, and 5.5 mM glucose) which was aerated with 5% CO_2_ in O_2_, thus maintaining a pH of 7.4 throughout the experiments. The temperature was kept at +37°C. All vessel segments were normalized to a transmural tension of 13.3 kPa/100 mmHg (*D*_100_) and the internal diameter of each vessel segment was then adjusted to 0.9 × *D*_100_.

The viability of each vessel segment was evaluated by substituting the buffer in the myograph bath with a potassium rich buffer (60 mM K^+^) causing prompt contraction. The mean force of two subsequent stimulations with a washout period of 5 min in between served as a measure of contractability for each vessel. Failure to produce a mean contractile force of >0.7 mN excluded the vessel segment from further experiments. There were no differences in contractile force between vessels in the tMCAO, SAH, and sham groups.

The endothelial function of each vessel segment was evaluated by measuring the dilatory response to carbachol (10^−5^ M) after precontraction with 5‐HT (3 × 10^−7^ M). Segments that failed to dilate >20% were excluded from further experiments. The number of viable vessel segments is reported along with the graphs below and only one segment was accepted from each rat so that n equals the number of rats used in each experiment.

Increasing concentrations of the P2Y_6_ agonist UDP‐β‐S were added to the vessel baths every 3 min in semi‐logarithmic steps (10^−11^–10^−4.5 M^). Force development was monitored continuously and recorded using the software LabChart (ADInstruments).

### 2.5 Immunohistochemistry

MCAs from male Wistar and Sprague‐Dawley rats (including tMCAO, SAH, and SAH‐sham) were carefully dissected from the brain as described above, and placed into TissueTEK (Gibo, Invitrogen S/S) and frozen. The embedded and frozen vessel specimens were then sectioned into 10 μm thick slices, fixed for 10 minutes in ice‐cold acetone (−20°C) and rehydrated in phosphate buffer solution (PBS, composition described above) containing 0.3% Triton X‐100 for 15 min before they were permeabilized and put in a blocking solution containing PBS, 0.3% Triton X‐100, 1% bovine serum albumin and 5% normal serum for 1 hour. The primary antibody (1:100, Alomone; #APR‐011) was diluted in PBS containing 0.3% Triton X‐100 and 1% bovine serum albumin and applied overnight at +4°C. Sections were subsequently incubated for 1h with the secondary antibody (FITC, goat anti‐rabbit, 1:100, Jackson ImmunoResearch, #111‐095‐003) diluted in PBS containing 0.3% Triton X‐100 and 1% bovine serum albumin before they were washed in PBS and mounted with Vectashield mounting medium (Vector Laboratories, Inc.). Immunoreactivity was visualized and recorded using a light‐ and epifluorescence microscope (Nikon 80i) at the appropriate wavelength. The same procedure as described above was used for the negative controls, except that either primary or secondary antibodies were omitted, resulting in no staining in the tissue except for auto‐fluorescence in the internal elastic lamina.

### 2.6 Flow cytometry

Vascular smooth muscle cells were isolated as described previously (Radziwon‐Balicka et al., 2017) by placing tissue from MCAs of tMCAO, SAH, and SAH‐sham rats in an enzymatic digestion solution containing highly purified collagenase I and collagenase II in a medium of Thermolysin (Liberase TM Research Grade; Roche) for 1.5 h at +37°C in a thermomixer (Eppendorf) at 500 g. To improve digestion of the tissue, the suspension was pipetted up and down every 30–40 min during the procedure. The digestion process was stopped by physiological buffered saline (PBS) containing 5% bovine serum albumin (BSA, Sigma Aldrich). Next, the samples were sieved through a 70 µm cell strainer to separate single cells and remove clusters of undigested tissue. The cell suspension was rinsed with ice‐cold PBS +5% BSA. Collected cells were centrifuged at 400 g for 10 min at room temperature and the cells were once again washed in PBS. 1 µl of Fixable Viability Dye eFluor 780 (FVD, eBioscience) was added to the cell samples before they were immediately vortexed and incubated for 30 min at +2–8°C, protected from light. The FVD is used to irreversibly label dead cells prior to fixation and permeabilization procedures. Finally, the isolated cell suspension was fixed with 4% paraformaldehyde (PFA) for 20–30 min and then the cells were washed with PBS for further analysis.

Intracellular flow‐cytometry was performed in order to investigate the expression of P2Y_6_ on VSMCs within MCAs. 1 ml of 0.25% Triton X‐100 (Sigma Aldrich) in PBS buffer was added to cells pellets in order to permeabilize the cell membranes. The samples were incubated for 30 min at room temperature with rocking, protected from light and washed once with 1 ml of PBS. Next, the cells were resuspended in blocking buffer containing 5% normal donkey serum in PBS and incubated for 2 h at room temperature with rocking, protected from light. The cell suspensions were stained overnight at +4°C temperature with primary goat anti‐SM22α (1:100, Abcam) and primary rabbit anti‐P2Y_6_ (1:100, Alomone), respectively or goat isotope control IgG (Abcam), rabbit isotope control IgG (Abcam) in blocking buffer (PBS + 5% normal donkey serum) protected from light with rocking, respectively. Excess primary antibodies were removed by washing with PBS. Subsequently, the cell samples were incubated with Alexa‐488 anti‐goat IgG (Life Technologies) and allophycocyanin (APC)‐conjugated donkey anti‐rabbit IgG (Jackson ImmunoResearch) for 2 h at room temperature, in the dark with rocking. Excess of secondary antibody was removed by washing with PBS and then diluted to a final volume of 0.3 ml with PBS before analysis by flow‐cytometry. The stained cells suspension was analyzed by fluorescent‐activated cell sorting (FACS) on the BD FACSVerse machine (BD Biosciences, USA). Fluorescence was excited with a 488 nm blue laser and 640 nm red laser. First the viable cells negative for FVD 780 were gated and a population of SM22α‐positive cells was identified. Next, a sub‐gate was set in order to detect P2Y_6_ receptor expression on SM22α‐positive cells. The ratio of SM22α‐positive cells expressing P2Y_6_ was calculated for each sample. Data was analyzed using the BD FACSuite software.

### 2.7 Data analysis

Vessel contraction is expressed as a percentage of the initial contraction induced by 60 mM K^+^. All concentration‐response curves were analyzed by iterative non‐linear regression analysis using GraphPad Prism 8.02 (GraphPad Corp). Sensitivity to agonists is expressed as −log EC_50_ (M) where EC_50_ [M] is the molar concentration of agonist required to produce half of the maximal response. Data are presented as median and [IQR] unless otherwise stated and all n equals the number of unique vessel segments in each experiment.

The Kruskal–Wallis test with post hoc Dunn's multiple comparison test was performed when comparing more than two groups. *p* < 0.05 were considered statistically significant. A power calculation was made using the G*power 3.1.9.2. A two‐tailed alpha of 0.05 was assumed along with a power of 80% to detect a 30% change in Emax.

## 3 RESULTS

### 3.1 Animals

The experiments in this paper are based upon 45 rats of which 15 underwent tMCAO, 14 rats underwent experimental SAH and 12 rats underwent sham surgery and survived until termination after 48 h. Two rats died during surgery and another five rats were terminated due to technical failure (no CBF drop) or animal distress. The number of viable segments used in the subsequent experiments and statistical analysis are presented in their respective graph and each segment represents a unique rat. Vessels from the sham treated animals were used as controls in all experiments and compared to vessel segments from SAH‐animals and tMCAO‐animals.

### 3.2 Endothelial function

Precontracted vessel segments failing to dilate >20% upon carbachol exposure were excluded from further experiments. There was no difference in dilatory response between the remaining vessel segments in the sham group, tMCAO, and SAH groups respectively (29.1 [23.7–33.4]% vs. 38.9 [23.8–66.0]% and 39.3 [23.6–68.7]%, ns).

### 3.3 Enhanced vasoconstrictive response to UDP‐β‐S after tMCAO

**FIGURE 1:**
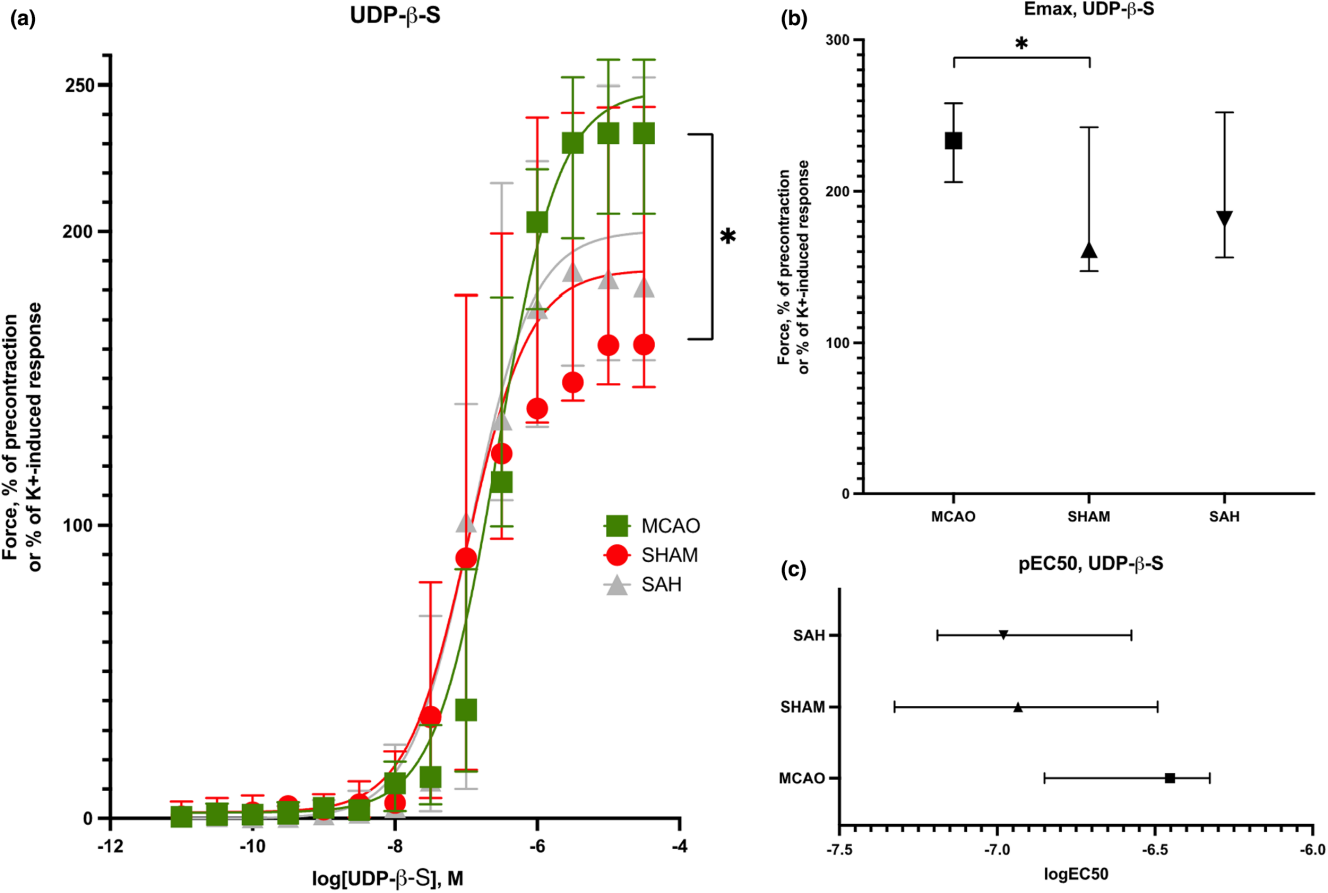
Wire myograph experiments demonstrating the vasoconstrictive actions by cumulative application of UDP‐β‐S on the middle cerebral artery following tMCAO (*n* = 10), SAH (*n* = 11) or sham surgery (*n* = 9) (a). E_max_ and pEC_50_ are presented in (b) and (c) respectively. Data are presented as median and IQR, *p < 0.05

The MCA segments in the tMCAO group (*n* = 10) and sham surgery group (*n* = 9) contracted in a concentration‐dependent manner when subjected to UDP‐β‐S (Figure 1). There was a significantly enhanced Emax for the tMCAO vessels compared to the sham vessels (233.6 [206.1–258.5]% vs. 161.1 [147.1–242.6]%, *p* = 0.0365). There was no difference in pEC50 (6.45 [6.33–6.85] vs. 6.93 [6.49–7.33], ns).

### 3.4 No alterations in vasoconstrictive response to UDP‐β‐S after SAH

The MCA segments from SAH animals (*n* = 11) and sham surgery animals (*n* = 9) contracted in a concentration‐dependent manner when subjected to UDP‐β‐S (Figure 1). No differences were seen in Emax (181.4 [156.2–252.5]% vs. 161.1 [147.1–242.6]%, ns) or pEC50 (6.98 [6.56–7.19] vs. 6.93 [6.49–7.33], ns) between the groups.

### 3.5 Enhanced expression of P2Y6 receptors in VSMCs after tMCAO and SAH

**FIGURE 2:**
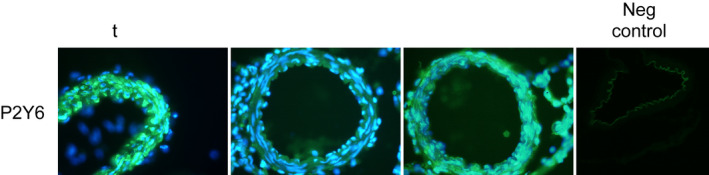
Confocal microscope images demonstrating expression of P2Y_6_ (FITC, green) in cross‐sections of middle cerebral arteries. DAPI (4′,6‐Diamidine‐2′‐phenylindole dihydrochloride, a blue nucleus stain, has been added. The tMCAO, Sham, and SAH images were generated by superimposing a DAPI stained image on a FITC stained image. The negative control demonstrates the lack of unspecific antibody binding and the autofluorescence of the lamina elastica interna which separates the intima from the underlying layer of smooth muscle cells

The immunohistochemistry revealed a strong presence of P2Y_6_ receptors on the cerebrovascular smooth muscle cells of the MCA (Figure 2). This was however, not visibly altered by tMCAO or SAH. In addition, the P2Y_6_ receptors could not be visualized on the endothelial cells of the MCA.

**FIGURE 3:**
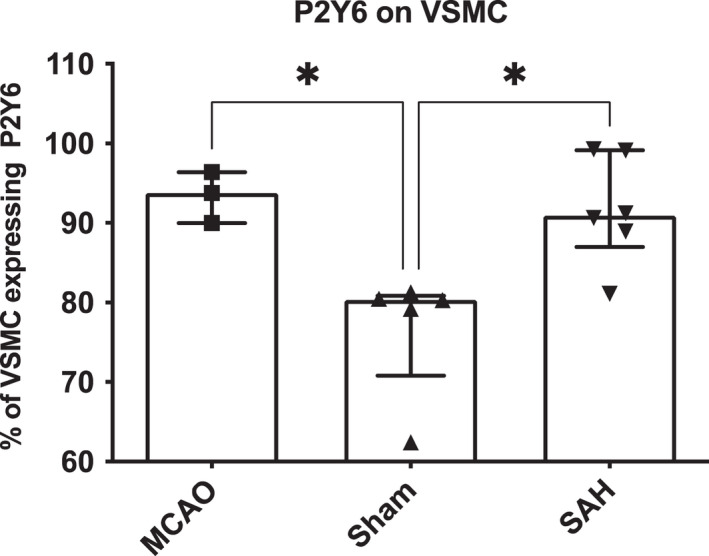
Bar graph with scatter plot demonstrating the expression of P2Y_6_ receptors on vascular smooth muscle cells determined by flow cytometry. Data are presented as median and IQR, *n* = 3–6, *p < 0.05

**FIGURE 4:**
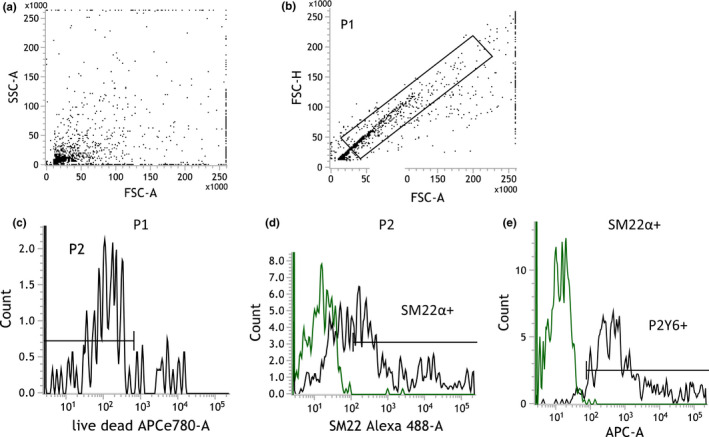
Representative dot plots and histograms of the experimental setup and analysis of flow cytometry data. (a) Dot plot histogram for entire cell population after isolation. (b) Dot plot histogram of single cells population according to FSC‐H (H—high) versus FSC‐A (A—area scaling). (c) Representative histogram of negative single cells population for Fixable Viability Dye eFluor 780 (viable cells, live/dead); (d) Representative histogram demonstrating SM22α‐positive events of viable cells suspension (log scale). (e) Representative histograms demonstrating P2Y_6_‐positive events of viable VSMC suspension (log scale)

Flow cytometry revealed increased expression of P2Y_6_ in the VSMCs after both SAH and tMCAO when compared to sham (90.94 [86.99–99.15]% and 93.79 [89.96–96.39]% vs. 80.31 [70.80–80.86]%, *p* = 0.021 and *p* = 0.039, respectively (Figures 3 and 4)).

## 4 DISCUSSION

This is the first demonstration of increased expression of P2Y_6_, a vasoconstrictor receptor belonging to the family of purine‐ and pyrimidine receptors, in cerebrovascular smooth muscle cells in the MCA following transient MCA occlusion. This was associated with increased vasoconstrictor responses following stimulation with UDP‐β‐S, a selective P2Y_6_ agonist. Experimental SAH also caused a significant increase in P2Y_6_ expression within the smooth muscle cells of the cerebral vasculature but the contractile responses to UDP‐β‐S remained unchanged.

Several papers have demonstrated the upregulation of a variety of vasoconstrictive receptors on the smooth muscle cells of cerebral arteries following an ischemic event (Edvinsson & Povlsen, 2011; Johansson et al., 2012; Müller et al., 2017; Stenman & Edvinsson, 2004). This increased expression of vasoconstrictive receptors is thought to contribute to cerebrovascular dysfunction, delayed ischemia, and/or vasospasm which worsens the outcome after both thromboembolic stroke and SAH.

Upregulation of vasoconstrictive receptor systems is associated with increased activity in the MEK‐ERK1/2 pathway (Edvinsson & Povlsen, 2011) and treatment with the MEK1/2 inhibitor U0126 seems to improve outcomes after SAH in animal models (Johansson et al., 2014; Povlsen & Edvinsson, 2015). Whether this holds true regarding the up‐regulation of P2Y_6_ receptors following tMCAO has to be further investigated.

The increased sensitivity to P2Y_6_‐mediated contraction after tMCAO is likely due to an increased expression of P2Y_6_ receptors on the vascular smooth muscle cells as demonstrated by our flow cytometry results. An additional, and possibly synergistic explanation is the partial destruction of the endothelial layer or increased permeability of the endothelial cells causing an increased exposure of P2Y_6_ receptors on smooth muscle to their ligand. The presence of P2Y_6_ receptors on the endothelium of the cerebral vasculature has been investigated and the current knowledge suggests that the endothelial cells within the cerebral vasculature of the rat contains predominantly P2Y_1_ and P2Y_2_ receptors. The lack of P2Y_6_ receptors on the endothelial cells is verified by immunohistochemistry in the present paper and is in line with the findings of Lewis et al who found no dilatory response of UDP on rat pial arteries (Lewis et al., 2000). Some destruction of the endothelial layer is inevitable during mounting in wire myographs, but vessel segments failing to dilate upon carbachol exposure were excluded and there was no difference in endothelial response when comparing tMCAO vessels or SAH vessels with sham vessels.

To further complicate things, there are reports of UDP having agonistic activity at the P2Y_14_ receptor in rats and antagonistic activity at the human P2Y_14_ receptor (Fricks et al.,). There are however no reports of either agonistic or antagonistic effects of UDP‐β‐S at the P2Y_14_ receptor in rats. Such actions cannot be ruled out and the results of this paper must be interpreted with this controversy in mind.

The physiological impact of increased signaling through smooth muscle P2Y_6_ receptors clearly indicates a trend towards increased vasoconstriction and reduced blood flow downstream of an ischemic lesion, especially if the endothelium is damaged. An interesting detail is the apparent involvement of P2Y_6_ receptors in the regulation of myogenic tone in cerebral arterioles by a mechanism that seems to be purely mechanical rather than auto‐ or paracrine (Brayden et al., 2013). Interestingly, there was no increase in vessel sensitivity to UDP‐β‐S following SAH despite increased expression of P2Y_6_ receptors on the smooth muscle cells. This may reflect a difference in the nature of the damage done to the blood vessels by tMCAO and SAH respectively.

The P2Y_6_ receptor seems a promising target for anti‐ischemic treatment given the profound increase in UDP‐mediated vasoconstriction following tMCAO. P2Y_6_‐blockade would also represent a new approach of inducing neuroprotection by targeting changes to the cerebral vasculature rather than a direct effect on neurons or clot formation. The increase in P2Y_6_ receptor density following both tMCAO and SAH may indicate a potential role of UDP‐mediated vasoconstriction in delayed ischemia after SAH as well. Vasospasm following aneurysmatic bleeding is a severe disorder and the role of the P2Y_6_ receptor under such conditions warrants further investigation.

Activation of purinergic receptors has also been linked to vascular inflammation and long‐term trophic events such as cell proliferation and differentiation associated with cardiovascular disease. UDP‐mediated signaling through P2Y_6_ receptors on microglia, for instance, seems to play an important role in the acute phase of ischemia and inflammatory states within the CNS (Anwar et al., 2020). Microglia express a variety of purinergic receptors, including P2Y_6_, which mediates microglial activation, cytokine secretion, and promotes phagocytosis of cellular debris (Koizumi et al., 2013; Mishra et al., 2016). The cytokines produced by microglia cause blood‐brain barrier disruption and tissue damage while also promoting neuronal survival through neurotrophic factors. Activated microglia may also promote short term neurological recovery by acting as scavengers handling cellular debris and apoptotic cells. Activation of P2Y_6_ receptors may stimulate ERK1/2 through activation of protein kinase C (Kim et al., 2003), a pathway previously shown to induce changes in several receptor systems favoring vasoconstriction (Müller et al., 2017). P2Y_6_‐mediated signaling has also been implied in activation of NF‐κB (Korcok et al., 2005), a pro‐inflammatory transcription factor which may both magnify inflammation and induce the resolution of an inflammatory state by enhancing leukocyte apoptosis (Lawrence, 2009).

It was recently demonstrated how the P2Y_6_ receptor antagonist MRS2578 increased infarct volumes and worsened outcomes after tMCAO (Wen et al., 2020) in mice, lending evidence to microglial phagocytosis being a beneficial process in the aftermath of cerebral ischemia. The overall effect of P2Y_6_ blockade thus seems detrimental in spite of the upregulation of P2Y_6_ receptors on VSMCs. Further investigation of the impact of increased vascular sensitivity to P2Y_6_‐mediated signaling and the effect of P2Y_6_‐mediated signaling in ischemia is warranted, ideally delineating the temporal course of changes in purinergic signaling that occur following ischemic events.

We have in this paper demonstrated increased vasoconstrictive effects of the P2Y_6_ agonist UDP‐β‐S on rat MCAs following tMCAO but not after experimental SAH. However, the VSMC content of P2Y_6_ receptor protein was significantly increased in both types of stroke.

## CONFLICT OF INTEREST

The authors declare no conflicts of interest.

## AUTHOR CONTRIBUTIONS

André Erdling performed wire myograph experiments, immunohistochemistry experiments, analyzed data, performed statistical analysis, prepared the figures, drafted, and finalized the manuscript. Sara Ellinor Johansson performed wire myograph experiments. Aneta Radziwon‐Balicka performed the flow cytometry experiments. Saema Ansar and Lars Edvinsson designed the study, supervised the experiments, and revised the manuscript. All authors read and approved the finalized manuscript before submission.

## ETHICS APPROVAL

All animal handling, experiments, and procedures were performed in accordance with both the ARRIVE guidelines and the European Community Council Directive (2010/63/EU) for Protection of Vertebrate Animals Used for Experimental and other Scientific Purposes. Furthermore, all experimental procedures were approved either by the Lund‐Malmö Institutional Ethics Committee under Swedish National Department of Agriculture (M153‐15 and M188‐12) or by the Danish Animal Experimentation Inspectorate (license no. 2011/561‐2025) depending on the site for the experiment.
